# A Non-classified Equine Disease in Montana

**Published:** 1896-04

**Authors:** Robert H. Bird

**Affiliations:** State Veterinarian, Helena, Montana


### A NON-CLASSIFIED DISEASE AFFECTING RANGE-HORSES IN MONTANA.

By ROBERT H. BIRD, M.R.C.V.S.,
STATE VETERINARIAN, HELENA, MONTANA.

THE perusal of an article in the February number of the JOURNAL by State Veterinarian Scott, of Wisconsin, under the heading "Golden-rod Killing Horses," will serve as a basis for a few brief remarks on the subject of a disease met with in Montana among horses, and which bears a striking similarity in its pathological characteristics to that described by the above-named gentleman.

For several years a disease has been known to affect horses grazing on the ranges or unenclosed native pasture-tracts of Montana. These ranges are situated at an altitude averaging anywhere from 3000 to 4000 feet above sea-level. In the main chain of the Rocky Mountains and its numerous spurs these ranges may be heavily timbered, but on the large plains or bench-lands stretching in an easterly direction timber may be entirely wanting. The botanical features of the plants and grasses growing in these regions are presumably similar to the flora found in other sections of the Rocky Mountain country at a like altitude. The native or bunch-grass, though not so common as in former years, still grows abundantly in many parts, and is exceedingly nutritious. Multitudinous plant-life also flourishes, and to some of these plants poisonous properties are popularly attributed. The golden-rod, while not so abundant as in States further east, can be found, I understand, more or less generally over the State; but on the ranges its growth is limited, and it certainly is not conspicuous. No suspicion has yet been directed to the golden-rod as the causative agent of the malady to be referred to. I observe that the *United States Dispensatory* refers to the varieties of *Solidago*, or golden-rod, found on this continent, and mentions its medicinal virtues as "aromatic, stimulant, and carminative;" but no mention is made of any properties inimical to animal life, which is the subject of this article.

The disease prevails to a greater extent during some seasons than others. It has occasionally assumed the dimensions of an epizoötic. It begins to make its appearance generally in the early autumn, and is more markedly frequent after a hot, dry summer, succeeded by fall rains. While certain sections of the country are regularly visited by the disease, still it is liable to miss one or more seasons, and it has frequently been noted that while horses accustomed to graze on a certain side or section of a range would contract the disease, horses on the other side or on ranges adjacent thereto would remain perfectly healthy, although there were no barriers intervening or anything to prevent the animals freely commingling, which no doubt they often did.

The subjoined notes on the clinical and necroscopical characters of the disease in question were taken in the fall of 1892, a season when the disease was very rife, and when I had more extended opportunities for observing it than have occurred to me at any subsequent season. The present winter in Montana is unprecedentedly mild, and several outbreaks of the disease have been recorded. The type, though not so virulent, remains much the same as seen in 1892.

Horses when first affected are observed in a drooping attitude and standing apart from the herd. There is stiffness of the hind-limbs and a dragging of the same, with an uncertain staggering gait, due evidently to want of muscular control or incoordinate voluntary movement. There is a plaiting of the hind-limbs, seemingly due to weakness in the lumbar and gluteal regions. This symptom is a very noticeable one, especially when the animal is seen from a little distance and moving on a hillside. It has been attributed by ordinary observers to "kidney disease." The body temperature in very acute cases ranges up to 105° or 106° F. The subjects in which such intense thermic activity is maintained for several days at the start frequently die at this stage, and in those cases in which the reactionary period is reached the subsequent prostration and debility are very marked. The fever is of a distinctly remittent type, and its exacerbations would seem to depend to a great extent upon any sudden lowering of weather temperature.

I have observed that cases which had been stabled and submitted to medical treatment, and which appeared to be convalescing nicely—the body temperature having subsided to the normal—would frequently be found after a sudden cold spell to be suffering from a relapse, with an accession of fever. These relapses would leave the patient weaker and weaker and more emaciated, till death would take place two or three months or even longer after the primary attack. In such chronic cases a remarkable pallidity of the conjunctival and nasal mucous membranes is an almost unfailing symptom, even though the temperature may register three or four degrees above the normal; pulse up to 120° or higher; the heart-beats being abnormally loud; the cardiac impulse strong, but the submaxillary pulse small and feeble; jugular regurgitation; œdema of the limbs and under the chest and abdomen. A pneumonia of subacute type frequently supervenes in the later stages, and is accompanied by all the distressing symptoms of that condition. Irritability of the kidneys and bladder may be evinced by repeated evacuations, and pregnant mares commonly abort. When first attacked, blood-blotches, or petechiæ, are sometimes seen on the mucous membranes of the eyes and nostrils, or there may be evidences of actual hemorrhage from the latter. The appetite, as a rule, is in abeyance during the fever-stage; at other times it is fairly good.

The post-mortem appearances are such as are generally associated with blood-diseases. In the case of horses which die early the blood is dark-colored; it may remain fluid, or is feebly coagulable, or the fibrin constituent will have been found to have separated itself into one or more long plugs, filling the aorta and large arterial trunks. In chronic cases the blood is thin, watery, and deficient in coloring matter; the lungs at their front lobe, or lower margins, œdematous or solidified; increased pericardial effusion, and there may be deep staining of the endocardial membrane. Blood-spots are sometimes seen on the exterior of the small intestines. The solid viscera generally, in chronic cases, are softened in texture and seem to be undergoing a breaking-down process. The spleen is frequently enlarged to three or four times its natural size, its pulp black, and on exposure to air it becomes tarry in appearance. In one case examined the membranes of the spinal cord were congested in the lumbar regions, with an increase in the subarachnoidal fluid. Microscopically the blood shows that the red-corpuscular element is greatly diminished, and in some cases almost entirely disintegrated.

I have several cases of the disease at present under observation and treatment. This latter is not, as a rule, in any but mild cases attended with satisfactory results. The various antiseptics, soda hyposulphite, potass. chlorate, quinine, arsenic, and many other agents have all been tried with varying degrees of success. It is only in the case of the more valuable breeds or broken horses which are unfortunate enough to contract the disease when turned for a season on range-pasture that it would repay the owner to attempt treatment. In the case of common unbroken range-horses individual treatment would be unprofitable as well as impracticable. While convalescence is not uncommon, I have rarely seen a well-developed case of the disease wholly recover; the weakness of the hind extremities persists even when the patient is otherwise apparently well.

It is difficult to estimate the mortality in any one season among horses in Montana from this disease. In former years bands of one hundred head or more have been sadly decimated, but of late it has not been so marked, and owing to the great depreciation in value of range-horses, pure and simple, and their unsalability at any price, the finding of a few dead horses at intervals does not excite any comment.

As in the case of Dr. Scott's obscure disease, anthrax was suspected, but bacteriological investigation failed to reveal the bacillus anthracis. There is no evidence that the disease is contagious; the infecting material would seem to be derived from the soil or food. Work-horses stabled and only turned out at intervals to graze have been known to become affected.

I have long entertained the idea that some parasitic fungus developed upon the grasses was the prime factor in developing the disease in question in Montana range-horses, and such an hypothesis is equally tenable in the case of the Wisconsin horses. In my first annual report to the State Board of Stock Commissioners, dated December 31, 1895, occurs the following extract referring to this disease: "Its clinical and necroscopical characters point to some pathogenic organism in the blood as the active agent, and this is in all probability developed upon and conveyed into the system by the food."

Much is yet to be learned regarding this disease as observed in this section of the Rocky Mountains, and should favorable opportunities occur it will form an interesting subject for experimentation and microscopical investigation at no distant date. It would be very interesting to learn if veterinarians in any other portions of the Rocky Mountain regions have observed a similar disease among horses.

